# Correlations between Hippocampal Neurogenesis and Metabolic Indices in Adult Nonhuman Primates

**DOI:** 10.1155/2011/875307

**Published:** 2011-08-02

**Authors:** Tarique D. Perera, Dunyue Lu, Lakshmi Thirumangalakudi, Eric L. P. Smith, Arkadiy Yaretskiy, Leonard A. Rosenblum, John G. Kral, Jeremy D. Coplan

**Affiliations:** ^1^Department of Psychiatry, College of Physicians and Surgeons, Columbia University, New York, NY, USA; ^2^Division of Geriatric Psychiatry, New York State Psychiatric Institute, New York, NY, USA; ^3^Department of Psychiatry, College of Physicians and Surgeons, Unit # 126, 1051 Riverside Drive, New York, NY 10032, USA; ^4^Department of Psychiatry, State University of New York Downstate Medical Center, Nonhuman Primate Facility, Department of Psychiatry, Brooklyn, NY, USA; ^5^Department of Surgery, SUNY Downstate Medical Center, Brooklyn, NY, USA

## Abstract

Increased neurogenesis in feeding centers of the murine hypothalamus is associated with weight loss in diet-induced obese rodents (Kokoeva et al., 2005 and Matrisciano et al., 2010), but this relationship has not been examined in other species. Postmortem hippocampal neurogenesis rates and premortem metabolic parameters were statistically analyzed in 8 chow-fed colony-reared adult bonnet macaques. Dentate gyrus neurogenesis, reflected by the immature neuronal marker, doublecortin (DCX), and expression of the antiapoptotic gene factor, B-cell lymphoma 2 (BCL-2), but not the precursor proliferation mitotic marker, Ki67, was inversely correlated with body weight and crown-rump length. DCX and BCL-2 each correlated positively with blood glucose level and lipid ratio (total cholesterol/high-density lipoprotein). This study demonstrates that markers of dentate gyrus neuroplasticity correlate with metabolic parameters in primates.

## 1. Introduction

The hippocampus is receiving increasing attention for its potential role in energy regulation [1]. The hippocampus is part of a neural circuit involved with reward and energy regulation [2] and is sensitive to satiety signals associated with learning and memory [3]. Recent findings indicate that palatable high-fat diets promote excessive food intake and weight gain and interfere with hippocampal functioning. This is supported by epidemiological data linking diets high in saturated fat with weight gain and memory deficits [4–6]. Furthermore, rats and humans with diabetes mellitus show age-related performance impairments on memory tasks [7]. More recent studies demonstrate that high-fat diet-induced maternal obesity impairs offspring hippocampal BDNF production [8], alters fetal hippocampal development [9], and reduces hippocampal neurogenesis during the early life of their offspring [10]. Moreover, adult male rats fed with a high-fat diet show impaired hippocampal neurogenesis [11]. Neurogenesis induced by ciliary neurotrophic factor (CNTF) or brain-derived neurotrophic factor (BDNF) in feeding centers of the murine hypothalamus is associated with weight loss in obese rodents [12, 13]. Because both hippocampal neurogenesis and proxy metabolic parameters are connected with stress and mood disorders [14–16], the aforementioned hypothalamic data raise important questions regarding the relationship between hippocampal neurogenesis and the regulation of peripheral metabolic parameters. We present a pilot study of the relationship between hippocampal neurogenesis and metabolic parameters in adult nonhuman primates. 

## 2. Methods

### 2.1. Subjects

All animal work has been conducted according to relevant national and international guidelines. In accordance with the recommendations of the Weatherall report “The use of nonhuman primates in research”, the following statement to this effect has been included to document the details of animal welfare and steps taken to ameliorate suffering in all work involving non-human primates. This work was conducted at the Nonhuman Primate Facility of the State University of New York Downstate Medical Center with permission from its Institutional Animal Care and Use Committee (IACUC).

### 2.2. Adverse Rearing Paradigm

Singly housed adult male bonnet macaques with a history of adverse rearing during infancy (*N* = 4) or normal rearing during infancy (*N* = 4) were matched for age and weight. The rearing conditions were established in previous studies [17, 18]. Adult subjects with a history of adverse rearing during infancy were those whose mothers were exposed to Variable Foraging Demand (VFD) conditions that involved alternating 2-week periods of easy and difficult food foraging conditions for a total of 16 weeks. The control subjects had mothers that were exposed to Low Foraging Demand (LFD) (control) conditions throughout this period. 

### 2.3. Morphometry

During anesthesia for blood sampling, weight in kilograms and crown-rump length (CRL) were measured, where CRL was the length in centimeters from the vertex of the head to the base of the tail. Measurements were consistently performed by the same team of investigators blinded to rearing condition as reported in an earlier study [19].

### 2.4. Blood Chemistry

Using previous methods [19], venous blood was drawn in plain nonheparinized tubes between 0800–1100 h after an overnight fast. On the day before blood sampling, food was withdrawn at 1600 h, and water remained available *ad libitum*. Monkeys were individually captured in carrying cages, placed in single-animal squeeze cages, and anesthetized with ketamine (10–15 mg/kg). Blood from antecubital or femoral veins was immediately placed on ice, centrifuged for serum separation, and stored at −80°C within 1 h. Samples were analyzed in one batch by routine laboratory procedures at the University Hospital of Brooklyn Clinical Chemistry Laboratory. Serum glucose was measured by glucose oxidase; serum triglycerides (TGs) were determined enzymatically using Ektachem Clinical Chemistry Slide; high-density lipoprotein (HDL), low-density lipoprotein (LDL), and total cholesterol were measured using the Vitros Chemistry Magnetic Reagent (Clinical Diagnostics Operator's Manual 1995, Johnson & Johnson, New Brunswick, NJ).

### 2.5. Neurohistochemistry

**Figure 1:**
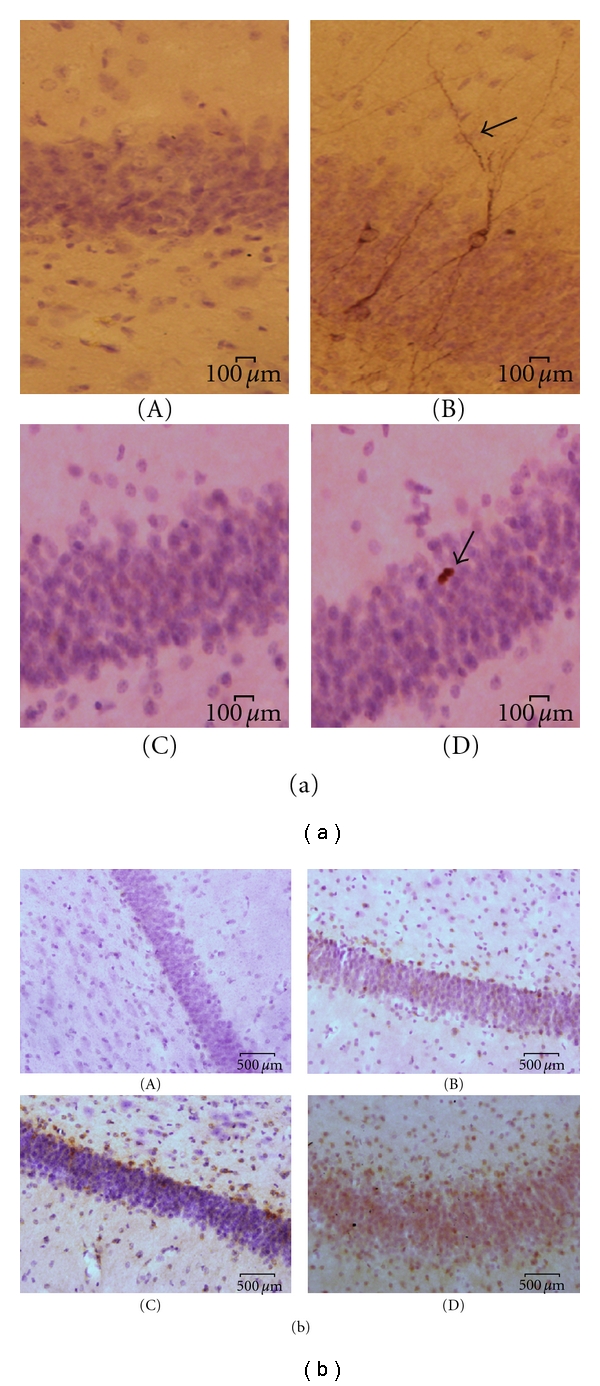
Representative images of histological studies of the dentate gyrus of hippocampus. (a) Granule cells in the subgranular zone (SGZ) positive (arrows) for DCX and Ki67. (A) represents negative control for DCX. (B) represents positive staining for DCX. (C) represents negative control for Ki67. (D) represents positive staining for Ki67. Scale bar: 100 *μ*m. (b) Images of BCL2-labeled cells in the SGZ. BCL2 gene expression in the SGZ was rated using a semiquantitative according to a 4-point scale. (A) represents no labeling; rated as 0. (B) represents light labeling of individual cells; rated as 1. (C) represents heavy, yet noncontinuous labeling of BCL-2 along the SGZ; rated as 2. (D) represents intense labeling forming a continuous band along the entire length of the SGZ; rated as 3. Scale bar: 500 *μ*m.

After obtaining blood samples and morphometry, all subjects were euthanized using established methods [14, 20]. Briefly, the subjects were anesthetized to a surgical depth with sodium pentothal and transcardially perfused with saline and formalin. The brains were removed and postfixed in 4% paraformaldehyde for immunnohistochemical staining and analysis. The left hippocampus was cut into 40 *μ*m sections. Every 40th section (approximately 10–12 sections per antibody) through the rostrocaudal extent of the left hippocampus was immunostained with standard peroxidase to detect and quantify cell proliferation and neurogenesis rates using our previous methods [14, 20]. Standard peroxidase methods were used to determine the following: granule cells proliferating at the time of sacrifice identified by the expression of the mitotic marker Ki67 (mouse anti-Ki67 antibody, 1 : 200; Vector Laboratories, Burlingame, CA) or B-cell chronic lymphocytic lymphoma 2 (BCL2) (anti-BCL2 (1 : 200); Dako, High Wycombe, UK); new neurons that were still immature, detected by the expression of the microtubule-associated protein doublecortin (DCX) [20] (goat anti-doublecortin, 1 : 200; Santa Cruz, CA). The secondary antibody is biotinylated anti-mouse IgG (1 : 200; Vector Laboratories, Burlingame, CA), and was visualized with avidin-biotin complex solution (Vector Laboratories, Burlingame, CA) and diaminobenzidine (DAB; Sigma-Aldrich, St Louis, MO). The density of DCX- or Ki67- labeled cells per mm^3^ of the SGZ was estimated for each animal. Expression of BCL2 in the SGZ was semiquantitatively graded using a previously established four-point rating scale method [14]. Two independent raters, masked to treatment condition, counted all of unambiguously Ki67 or DCX-labeled cells in the subgranular zone (SGZ) of the dentate gyrus (defined as a two-cell-body-wide zone on either side of the border of the granule cell layer) using a 40x objective (Figure 1(a)). 

### 2.6. Quantification and Statistical Analyses

Only DCX expressing cells that had tertiary dendrites were counted (Figure 1(a) panel B). Based on established methods [14], the density of Ki67 or DCX labeled cells was calculated. The density was defined as the number of positively stained cells divided by the volume of the SGZ. The volume of the SGZ was calculated as the length of the SGZ (per section) multiplied by the thickness of each section (40 *μ*m) multiplied by approximate width of two cells (50 *μ*m). 

Given the diffuse nature of BCL2 labeling, a previously established 4-point semiquantitative rating scale was used to evaluate the extent of BCL2 expression [14]. BCL2 gene expression in the SGZ was rated according to the following: (A) represents no labeling; rated as 0. (B) represents light labeling of individual cells; rated as 1. (C) represents heavy, yet noncontinuous labeling of BCL-2 along the SGZ; rated as 2. (D) represents intense labeling forming a continuous band along the entire length of the SGZ; rated as 3. 

Pearson correlation analyses were used to elucidate the relation among morphometric indices (body weight, crown-rump length), metabolic parameters (blood glucose and plasma lipids and lipid ratio), and neurogenesis markers (dentate gyrus DCX, BCL-2 and Ki67). A general linear model was used to assess whether the effects of body mass were independent of age. 

## 3. Results

The age range for all subjects was 9 to 12 years old with VFD-reared subjects (mean age 11 yrs, SD = 1.41) and LFD-reared subjects (mean age 11 yrs SD = 1.41) showing no difference (df = 6, *P* = 1.0). The weight range was 5.5 to 14 kg with VFD-reared (mean weight 11.5 kg, SD = 2.79) and LFD-reared subjects (mean weight 9.37 kg, SD = 2.90) showing no difference (df = 6; *P* = 0.33). Table 1 shows means and standard deviations of individual parameters for all subjects, and Figure 1 displays representative images of DCX, BCL-2, and Ki67 positive cells.

**Figure 2:**
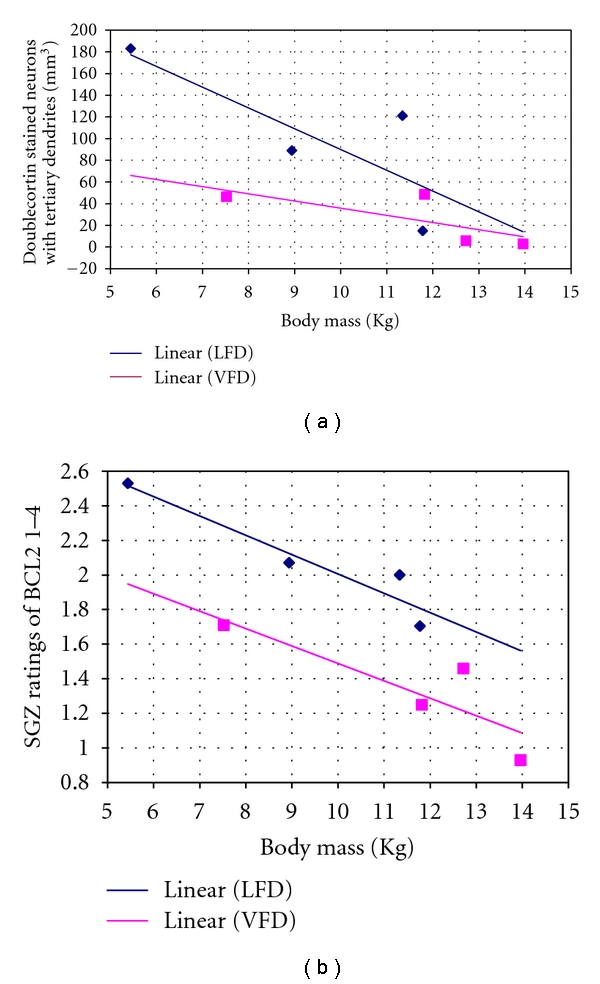
Correlation between *in vivo* adult body mass and postmortem measures of neurogenesis levels and BCL2 Expression. (a) shows a significant inverse correlation (*r* = −0.75, *P* = 0.03, *n* = 8) between body mass (in kilograms) and neurogenesis rates (indicated by the density of DCX-stained neurons with tertiary dendrites in per mm^3^ in the SGZ). Regression line of both groups combined not shown. (b) shows a significant, inverse correlation (*r* = −0.73, *P* = 0.041, *n* = 8) between body mass (in kilograms) and BCL2 expression in the SGZ (determined by a 4-point rating scale). Regression line of both groups combined not shown. These results show that animals with higher body mass have reduced neurogenesis rates and BCL-2. DCX: doublecortin; BCL-2: B-cell leukemia lymphoma 2. VFD: variable foraging demand (adversely-reared); LFD: low foraging demand (normally reared).

There was a significantly inverse correlation of body weight with the number of DCX-stained neurons with tertiary dendrites (*r* = −0.76, *P* = 0.03, *n* = 8) and BCL2-ratings of density (*r* = −0.73, *P* = 0.041, *n* = 8) (Figure 2). Using a general linear model, it was demonstrated that the relationship between DCX with tertiary dendrites and weight remained all but significant [F(1 : 5) = 6.34; *P* = 0.053] for both LFD and VFD reared animals when adjusted for age [F(1 : 5) = 1.18; *P* = 0.32]. Furthermore, BCL-2 expression correlated with body weight [F(1 : 5) = 21.05; *P* = 0.006] for both LFD and VFD reared animals, when adjusted for age [F(1 : 5) = 5.46; *P* = 0.067]. These findings indicate that neuroplastic effects in the dentate gyrus were dependent upon a relationship to body mass, which was independent of the effects of age. There were also significant inverse correlations between the crown-rump length and the number of DCX-stained neurons with tertiary dendrites (*r* = −0.98; *P* = 0.02, *n* = 4) and BCL2-ratings of density (*r* = −0.99; *P* = 0.006, *n* = 4) (Table 2). In addition, the fasting serum glucose levels were positively correlated with the DCX-stained neurons with tertiary dendrites (*r* = 0.97; *P* = 0.021, *n* = 4) and the BCL2-ratings of density (*r* = 0.99; *P* = 0.01, *n* = 4) (Table 2). The same was true for the blood lipid ratio, but not individual lipid parameters, which also positively correlated with DCX (*r* = 0.96; *P* = 0.042, *n* = 4), but only at trend levels for BCL-2 (Table 2). There was no significant correlation between the cell proliferation marker, Ki67, and metabolic parameters. 

## 4. Discussion

To our knowledge, this is the first report to show that markers of dentate gyrus neuroplasticity correlated with metabolic parameters. However, precursor proliferation, as indicated by Ki67, did not correlate with the metabolic parameters. The results suggest that neurogenesis, but not precursor proliferation, is associated with metabolic processes. Indicating that the findings of the current study were not an artifact of a possible relationship between body weight and central parameters, our previous monkey MRI study revealed no relationship between grey matter volume and body weight [21].

Studies in rodents demonstrate that high-fat diet causing weight gain impairs hippocampal neurogenesis through increased lipid peroxidation associated with decreased BDNF in the hippocampus [22, 23] and/or through glucocorticoid-mediated effects on immature or mature new neurons [24, 25]. In mice, genetic models deficient of BDNF signaling exhibit hyperphagia and obesity [26] in addition to suppressing hippopcampal neurogenesis [27]. In humans, obese children and adolescents exhibit reduced serum BDNF levels relative to their normal weight counterparts [28, 29]. Both central and peripheral administration of BDNF decrease food intake, increase energy expenditure, and ameliorate hyperglycemia in diabetic mice by a central nervous-system-mediated mechanism [25, 28, 30]. The antidepressant effects of BDNF infusion are closely linked to the induction of hippocampal neurogenesis [22]. Indeed, BDNF plays an important role in regulation of neurogenesis, angiogenesis, and synaptogenesis in the hippocampus [22, 31]. Therefore, BDNF may be one of the common pathways mediating both neurogenesis in the hippocampus and systemic metabolic process, such as insulin resistance. Together these preclinical and clinical findings imply an inverse correlation between hippocampal neurogenesis and body weight. 

Our data also showed that the hippocampal neurogenesis correlated positively with blood glucose level and lipid ratio, indicating that increased neurogenesis is associated with elevated metabolic activity [31]. Under physiological conditions, adult neurogenesis occurs within an angiogenic niche—a microenviroment, providing metabolic support for neurogenesis—within the hippocampus. The microenvironment is modulated by the circulating system that supplies mesenchymal stem cells and nutrients (e.g., glucose) [31]. Specific mechanisms linking hippocampal neurogenesis interacting with the hypothalamus and regulating appetite and intake behavior remain unclear. 

One potential candidate for the current link between hippocampal neurogenesis and metabolic markers is leptin. Leptin is a hormone secreted by adipose tissue and has effects at hypothalamic receptors that control food intake. Deficits in leptin or leptin receptors result in obesity, indicating the importance of the hormone in body mass homeostasis [32]. Leptin levels positively correlate with fasting insulin, fasting glucose, and triglycerides in nondiabetic men [33]. Studies show leptin increases adult hippocampal neurogenesis mainly by increasing cell proliferation and not by promoting cell differentiation and survival and reduces body weight (inversely correlated with cell differentiation) of animals [34]. By contrast, our study demonstrated that weight was inversely correlated with differentiation, as reflected by the generation of new neurons and survival, indicated by BCL-2 expression, but there was no correlation between weight and cell proliferation. The discrepancy between our findings and others may be due to multiple factors, such as differences in species, studies, and interventions. 

To our knowledge, this is the first study to show correlation between neurogenesis and metabolic parameters in nonhuman primates. There are several limitations of this study, including the small sample size and the inability to reveal a causal link between central and metabolic process. Further studies should investigate the relationship between neuroplasticity and peripheral metabolic processes. 

## Figures and Tables

**Table:** (a) Histological findings in subjects exposed to adverse rearing (VFD) and normal control conditions (LFDs)

Subjects	VFD			LFD controls			Statistics		
Parameter	*N*	Mean	Std Dev	*N*	Mean	Std Dev	*t*-value	df	*P*-value
Age (year)	4	11	1.41	4	11	.41	0	6	1
Weight (kg)	4	11.51	2.8	4	9.38	2.91	−1.06	6	0.332
Ki67 expressing cells (per mm^3^ of SGZ)	4	22.7	7.84	4	43.64	9.32	3.44	6	0.014
DCX expressing cells (per mm^3^ of SGZ)	4	75.43	96.42	4	262.5	116.33	2.48	6	0.048
BCL2 ratings (grades 1–4)	4	1.34	0.33	4	2.08	0.34	3.11	6	0.021

**Table:** (b) Metabolic parameters in the subgroup of subjects

Parameter	*N*	Mean	Std Dev
Glucose (mg/mL)	4	76.50	1.73
Crown Rump Length (cm)	4	19.81	1.28
Cholesterol (mg/dL)	4	117.50	21.23
Triglyceride (mg/dL)	4	50.00	20.03
HDL (mg/dL)	4	45.75	8.61
LDL (mg/dL)	4	61.75	22.20
Cholesterol/HDL ratio	4	2.63	0.74

**Table 2:** Pearson's correlations between neurogenesis-related parameters in the dentate gyrus of the hippocampus and *in vivo* metabolic parameters (significant at *P* < 0.05).

	BCL-2	Ki67	DCX
Glucose	.99	.28	.99
	*P* = 0.001	*P* = 0.71	*P* = 0.01
Cholesterol	.28	−.61	.46
	*P* = 0.71	*P* = 0.38	*P* = 0.53
Triglyceride	−.39	−.05	−.30
	*P* = 0.60	*P* = 0.94	*P* = 0.69
HDL	−.88	−.31	−.81
	*P* = 0.11	*P* = 0.68	*P* = 0.18
LDL	.69	−.45	.81
	*P* = 0.30	*P* = 0.54	*P* = 0.18
Ratio (total Cholesterol/HDL)	.89	−.15	.96
	*P* = 0.10	*P* = 0.84	*P* = 0.03
Crown-Rump	−.99	−.21	−.97
	*P* = 0.006	*P* = 0.78	*P* = 0.02

Significant findings are bolded. There was no significant correlation between cell proliferation marker (Ki67) and metabolic parameters. DCX: doublecortin; BCL-2: B-cell lymphoma 2; HDL: high-density lipoprotein; LDL: low-density lipoprotein.
